# Expression and clinicopathological significance of KAI1, MACC1, and AGR2 in cervical squamous cell carcinoma

**DOI:** 10.1097/MD.0000000000045992

**Published:** 2025-11-14

**Authors:** Siyuan Wang, Pengfeng Zhu

**Affiliations:** a ChangZhou Maternal and Child Health Care Hospital, ChangZhou Medical Center, Nanjing Medical University, Jiangsu, China.

**Keywords:** AGR2, KAI1, MACC1, prognosis, squamous cell carcinoma of cervix

## Abstract

Colon cancer metastasis-associated protein 1 (MACC1) was initially identified in colorectal cancer and is linked to metastasis and prognosis in various malignant neoplasms. Anterior gradient homology protein 2 (AGR2) has emerged as a significant prognostic indicator for multiple cancers, while anti-cancer 1 (KAI1), recognized as a tumor metastasis suppressor gene, plays a critical role in cancer progression. Despite their individual associations with metastasis and prognosis across different cancer types, the interactions among MACC1, AGR2, and KAI1 specifically in cervical squamous cell carcinoma remain poorly understood. Therefore, this study aimed to investigate the relationships between MACC1, AGR2, and KAI1 within the context of cervical squamous cell carcinoma, while also examining their correlations with clinicopathological features and overall survival (OS) outcomes in affected patients. Immunohistochemistry was used to detect the expression of MACC1, AGR2, and KAI1 in 106 cases of cervical squamous cell carcinoma. At the same time, the clinicopathological parameters and postoperative survival information were collected. In cervical squamous cell carcinoma, the detection rates of MACC1 and AGR2 were found to be significantly elevated, whereas the detection rate of KAI1 was notably reduced when compared to control tissues. Furthermore, the positive detection rates of MACC1 and AGR2 exhibited a positive correlation with tumor grade, tumor-node metastasis classification, and lymph node metastasis (LNM) stage, while demonstrating a negative correlation with OS. Conversely, the positive detection rate of KAI1 was negatively associated with tumor grade, tumor-node metastasis classification, and LNM stage. Patients within the KAI1 positive subgroup experienced a significantly extended OS in comparison to those in the KAI1 negative subgroup. Additionally, multivariate analysis indicated that the positive expression of MACC1, AGR2, and KAI1, alongside tumor stage and LNM stage, could serve as independent prognostic indicators for OS in individuals diagnosed with cervical squamous cell carcinoma. MACC1, AGR2, and KAI1 may represent potential metastatic and prognostic biomarkers, as well as promising therapeutic targets for squamous cell carcinoma of the cervix.

## 1. Introduction

Cervical cancer is a malignancy that arises in the cervix of females. The 2020 Global Cancer Statistics Report indicates that cervical cancer ranks as the 4th most prevalent and fatal cancer among women globally. In 36 countries, it is identified as the leading cause of cancer-related mortality among females.^[1,2]^ Despite China’s vigorous efforts in promoting preventive measures and control strategies against cervical cancer, the age-standardized incidence and mortality rates continue to rise annually, particularly in comparison to developed nations. Furthermore, high-risk strains of human papillomavirus are significantly implicated in the etiology of squamous cell carcinoma of the cervix, with human papillomavirus-related cases constituting over 90% of all instances.^[3,4]^ The asymptomatic nature of squamous cell carcinoma during its initial stages often results in late diagnoses, with many patients in China presenting at advanced tumor-node metastasis (TNM) stages III to IVA. This progression, along with tumor metastasis, is a critical factor contributing to the unfavorable prognoses observed in these patients.^[5,6]^

Anti-cancer 1 (KAI1), commonly referred to as CD82, has emerged as a pivotal tumor metastasis suppressor gene in recent research endeavors, initially recognized for its specific role in inhibiting metastasis in prostate cancer cells. The KAI1 gene is classified within the transmembrane 4 superfamily and is situated on chromosome 11P11.2. This gene plays a critical role in modulating the structure of the cell membrane through its interactions with integrins and other proteins belonging to the transmembrane 4 superfamily.^[7]^ Furthermore, KAI1 has been shown to impede the process of epithelial–mesenchymal transition (EMT) in cancer cells, thereby contributing to its biological functions.^[8]^ It also influences the Wnt/β-catenin signaling pathway, which serves to enhance cellular adhesion, stabilize the E-Cadherin/β-catenin complex, and prevent the detachment of cancer cells from the primary tumor site. Notably, KAI1 has been found to counteract the stimulatory effect of β-catenin’s tyrosine phosphorylation on hepatocyte growth factor (HGF).^[9]^ A growing body of evidence indicates that the down-regulation or complete loss of KAI1 expression is a significant factor promoting tumor metastasis and is closely linked to unfavorable prognoses across various forms of human malignancies.^[10]^

Colon cancer metastasis-associated protein 1 (MACC1) was 1st identified in colon cancer in 2009 and is recognized as a significant biomarker for both metastasis and prognosis in this malignancy.^[11]^ MACC1 plays a crucial role in modulating the transcriptional activity of the MET gene, serving as a principal regulator of the HGF/MET signaling pathway.^[12]^ Experimental studies have demonstrated that MACC1 enhances tumor cell proliferation, migration, and invasion in vitro, as well as promoting tumor invasion and metastasis in vivo.^[13]^ Beyond its pivotal role in colon cancer prognosis and metastasis, an increasing body of research indicates that MACC1 is also a valuable biomarker for a variety of other tumors.^[14]^

The human homolog of anterior gradient-2 (AGR2), which encodes a secreted protein, was initially discovered in *Xenopus laevis*.^[15]^ AGR2 belongs to the disulfide isomerase family, specifically the protein disulfide isomerase family, localized within the endoplasmic reticulum. As a protein disulfide isomerase, AGR2 is instrumental in the folding and maturation of receptor proteins.^[16]^ Furthermore, AGR2 functions as an oncogenic protein found in numerous cancers, where it modulates p53-related signaling pathways and promotes the expression of EGFR ligand proteins, thereby enhancing cell survival and stimulating the growth of cancer cells.^[17]^ AGR2 is often overexpressed in various malignancies, including breast, oral, lung, prostate, and pancreatic cancers, and serves as a reliable biomarker for prognosis in clinical settings.^[18]^

In summary, extensive research on KAI1, MACC1, and AGR2 has illustrated their involvement in tumor invasion and metastasis. However, investigations into the expression levels of KAI1, MACC1, and AGR2 in cervical squamous cell carcinoma, the interrelations among these biomarkers, and their associations with clinicopathological factors remain sparse. This study aims to evaluate the expression of KAI1, MACC1, and AGR2 in cervical squamous cell carcinoma, to explore the hypothesis of their interconnectivity, and to analyze their correlations with various clinicopathological factors, including metastasis and prognosis in cervical squamous cell carcinoma.

## 2. Materials and methods

### 2.1. Clinical data

Tissue wax blocks of cervical squamous cell carcinoma, well-preserved, were obtained from the ChangZhou Maternal and Child Health Care Hospital, with cases confirmed through pathological diagnosis between January 2018 and December 2020. A total of 106 cases were incorporated into this investigation, all of which had comprehensive clinical histories, pathological diagnoses, and follow-up data. Concurrently, 106 adjacent non-tumor cervical tissues from the same patients served as control specimens. Cases that underwent any form of preoperative anti-cancer treatment, including chemotherapy or radiation therapy, were excluded from the analysis. The overall survival (OS) was determined based on data collected from the 106 selected patients, monitored until their date of death or until December 2024, with a mean survival duration of 55.1 months, ranging from 19 to 78 months. This study was reviewed and approved by the Medical Research Ethics Committee of Changzhou Maternal and Child Health Hospital Affiliated to Nanjing Medical University.

Among the 106 cases of cervical squamous cell carcinoma, 41 patients were under the age of 60, while 65 patients were aged 60 or older. A smoking history was present in 25 cases, whereas 81 cases reported no such history. Regarding alcohol consumption, 36 patients had a history of drinking, and 70 patients did not. Concerning tumor size, 48 patients exhibited tumors smaller than 4.0 cm, while 58 patients had tumors measuring 4.0 cm or larger. In terms of lymph node metastasis, there were 68 patients classified as N0 and 38 patients as N1. The differentiation of tumors was categorized according to the World Health Organization criteria, revealing 42 highly differentiated tumors, 51 moderately differentiated tumors, and 13 poorly differentiated tumors. Tumor-node-metastasis (TNM) stage and lymph node metastasis stage were evaluated in accordance with the 7th edition of the American Joint Committee on Cancer guidelines. Specifically, TNM staging indicated that there were 32 cases classified as stage I, 35 cases as stage II, 36 cases as stage III, and 3 cases as stage IV. Detailed characteristics can be found in Table 1.

**Table 1:** Clinicopathological characteristics of the patients.

Clinicopathological features	Frequency (n)	Percentage (%)
Age		
<60	41	38.7
≥60	65	61.3
Tumor size (cm)		
<4.0	48	45.3
≥4.0	58	54.7
Smoking		
No	81	76.4
Yes	25	23.6
Drink		
No	70	66.0
Yes	36	34.0
Tumor grade		
High	42	39.6
Moderate	51	48.1
Low	13	12.3
Lymphatic metastasis		
N0	68	64.2
N1	38	35.8
TNM stage		
I	32	30.2
II	35	33.1
III	36	33.9
IV	3	2.8

TNM = tumor-node metastasis.

### 2.2. Immunohistochemical staining

Staining was performed using the Elivision TM Plus method according to the instructions of the purchased kit (Lab Vision, USA).

### 2.3. Criteria for judgment and evaluation of positive results

The interpretation of immunohistochemical staining results was conducted by 2 seasoned pathologists in a double-blind and independent manner. KAI1-positive cells were predominantly found within the cytoplasm and on the cell membrane, while MACC1-positive cells were largely situated in the cytoplasm, and AGR2-positive cells were primarily localized in the nucleus. A minimum of 10 representative high-power fields (magnification 400×) were examined microscopically, followed by scoring based on both the intensity of staining and the percentage of positively stained cells. The scoring system for staining intensity was defined as follows: no visible staining received 1 point; light yellow staining was awarded 2 points; brownish-yellow staining scored 3 points; and brown staining accounted for 4 points. For the percentage of positive cells, the scoring criteria were as follows: negative yielded 0 points; positive cells comprising <11% were given 1 point; positive cells between 10% and 51% scored 2 points; positive cells ranging from 50% to 76% received 3 points; and positive cells exceeding 75% were assigned 4 points. The cumulative score was obtained by multiplying the intensity score by the percentage score, yielding a final range from 0 to 12. A score exceeding 2 was classified as a positive outcome.

### 2.4. Statistical methods

The association between clinicopathological characteristics and the expression levels of KAI1, MACC1, and AGR2 was evaluated using either the Chi-square test or Fisher exact test. To investigate the interrelationships among KAI1, MACC1, and AGR2, the Spearman correlation coefficient was employed. The impact of KAI1, MACC1, and AGR2 on OS was assessed through both univariate and multivariate Cox regression analyses. Survival outcomes were analyzed using Kaplan–Meier and Log-rank methods, which facilitated the exploration of the relationship between immunohistochemical staining results of KAI1, MACC1, and AGR2, along with various clinicopathological factors and postoperative survival duration. The correlations involving KAI1, MACC1, and AGR2 staining outcomes, as well as clinicopathological characteristics, were computed using SPSS version 25.0 software for Windows (Chicago). A *P*-value of <.05 was deemed to indicate statistical significance.

## 3. Result

### 3.1. The expressions of KAI1, MACC1, and AGR2 were correlated with clinicopathological features

#### 3.1.1. Correlation between the expression of KAI1 in tissues and clinicopathological factors

**Figure 1.**
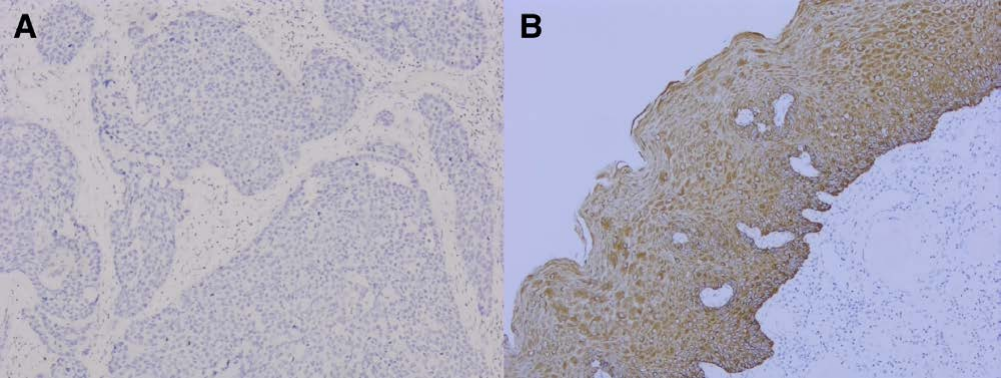
Expression of KAI1 in cervical squamous cell carcinoma and corresponding non-tumor control tissues. (A) Negative expression of KAI1 in cervical squamous cell carcinoma; (B) KAI1 is positively expressed in normal squamous epithelium. KAI1 = anti-cancer 1.

In a cohort of 106 diagnosed cases of cervical squamous cell carcinoma, KAI1 immunohistochemical staining was found to be positive in 42 cases (42/106, 39.6%). In contrast, the corresponding non-tumor tissues exhibited a significantly higher positivity rate, with 100 cases (100/106, 94.3%) showing KAI1 positivity (*P* < .05), indicating a statistically significant difference (refer to Fig. 1A and B). Within the same cohort, KAI1 expression was detected in 14 out of 41 cases (34.1%) in individuals younger than 60 years, and in 28 out of 65 cases (43.1%) in those aged 60 years or older. However, the difference between these 2 age groups was not statistically significant (*χ*^2^ = 0.838, *P* = .360). Further analysis revealed that KAI1 expression was positive in 26 of 48 cases (54.2%) in the group with tumor sizes <4.0 cm, while only 16 of 58 cases (27.6%) with tumors measuring 4.0 cm or greater showed KAI1 positivity, a difference that was statistically significant (*χ*^2^ = 7.757, *P* = .005). Regarding smoking status, KAI1 expression was noted in 32 of 81 nonsmokers (39.5%) and 10 of 25 smokers (40.0%), with no significant difference observed between these groups (*χ*^2^ = 0.002, *P* = .964). When examining drinking history, KAI1 positivity was found in 30 out of 70 cases (42.9%) in individuals without a history of alcohol consumption, compared to 12 out of 36 cases (33.3%) in those with such a history; the difference was not statistically significant (*χ*^2^ = 1.210, *P* = .271). When differentiating by histological grade, KAI1 positivity was present in 24 of 42 well-differentiated tumors (57.1%) and in 17 of 51 moderately differentiated tumors (33.3%). Notably, only 1 of the 13 poorly differentiated tumors (7.7%) exhibited KAI1 expression, with a significant difference observed across differentiation grades (*χ*^2^ = 11.773, *P* = .003). Lymph node metastasis, which reflects the tumor’s biological behavior, demonstrated KAI1 positivity in 38 of 68 cases (55.9%) in the N0 group, whereas only 5 of 38 cases (13.2%) in the N1 group were positive, indicating a statistically significant difference in KAI1 expression related to lymph node status (*χ*^2^ = 18.191, *P* < .001). Additionally, TNM staging, which is crucial for determining treatment and prognosis, was also associated with KAI1 expression. In stage I, 30 out of 34 cases (88.2%) were positive for KAI1, while in stage II, only 10 of 35 cases (28.6%) were positive, and no positive expression was detected in 3 cases of stage IV. The differences in KAI1 expression across TNM stages were statistically significant (*χ*^2^ = 46.650, *P* < .001). The detailed statistical outcomes are summarized in Table 2.

**Table 2:** Expression of KAI1 in cervical squamous cell carcinoma and its correlation with clinicopathological factors.

Group	KAI1		*χ* ^2^	*P*
	Negative	Positive		
Age			0.838	.360
<60	27	14		
≥60	37	28		
Tumor size (cm)			7.575	.005
<4.0	22	26		
≥4.0	42	16		
Smoking			0.002	.964
No	49	32		
Yes	15	10		
Drink			1.210	.271
No	40	30		
Yes	24	12		
Tumor grade			11.773	.003
High	18	24		
Moderate	34	17		
Low	12	1		
Lymphatic metastasis			18.191	<.001
N0	30	38		
N1	33	5		
TNM stage			46.650	<.001
I	4	30		
II	25	10		
III	32	4		
IV	3	0		

KAI1 = anti-cancer 1, TNM = tumor-node metastasis.

#### 3.1.2. Correlation between the expression of MACC1 in tissues and clinicopathological factors

**Figure 2.**
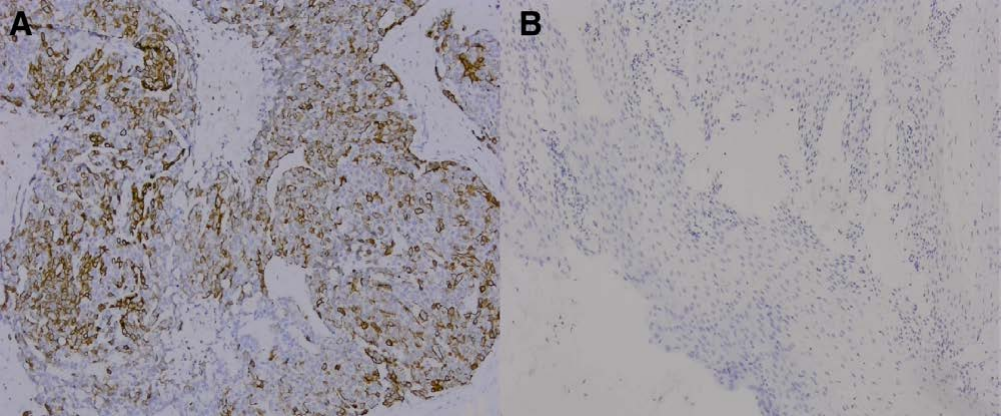
Expression of MACC1 in cervical squamous cell carcinoma and corresponding non-tumor control tissues. (A) Positive expression of MACC1 in cervical squamous cell carcinoma; (B) MACC1 is negatively expressed in normal squamous epithelium. MACC1 = colon cancer metastasis-associated protein 1.

Sixty-five of the 106 cases of squamous cell carcinoma of the cervix (65/106, 61.3%) were positive for MACC1, and 8 (8/106, 7.5%) of the corresponding non-tumor control tissues were positive for MACC1, *P* < .05. The difference in expression between the 2 groups was statistically significant (Fig. 2A and B). Among 106 selected cases of cervical squamous cell carcinoma, MACC1 expression was positive in 26 of 41 cases younger than 60 years of age (26/41, 63.4%) and in 39 of 65 cases older than or equal to 60 years of age (39/65, 60.0%). There was no significant difference between the 2 groups (*χ*^2^ = 0.124, *P* = .725). In the tumor diameter group, 27 out of 48 cases (27/48, 56.3%) were positive for MACC1, and 38 out of 58 cases (38/58, 65.5%) were positive for MACC1 in the ≥ 4.0 cm group (*P* = .329); in the group with and without smoking history, MACC1 expression was positive in 47 of 81 (47/81, 58.0%) cases in the nonsmoking group and in 18 of 25 (18/25, 72.0%) cases in the smoking history group, and there was no significant difference in the smoking history group (*χ*^2^ = 2.772, *P* = .100). In the group with and without drinking history, 43 cases (43/70, 61.4%) were positive for MACC1 in the group without drinking history, and 22 cases (22/36, 61.1%) were positive in the group with drinking history. There was no significant difference in the expression of MACC1 in the group with drinking history (*χ*^2^ = 0.001, *P* = 1.000). The expression of MACC1 was significantly correlated with the degree of tumor differentiation. The expression of MACC1 was positive in 16 of 42 (16/42, 38.1%) well-differentiated tumors and in 36 of 51 (36/51, 70.6%) moderately differentiated tumors. MACC1 expression was positive in 13 cases of differential differentiation group (13/13, 100%), and there was a significant difference in the expression of MACC1 between different differentiation groups (*χ*^2^ = 19.599, *P* < .001). The expression of MACC1 was positive in 33 of 68 cases (33/68, 48.5%) in N0 group and 32 of 38 cases (32/38, 84.2%) in N1 group. The expression of MACC1 was significantly different in lymph node metastasis group (*χ*^2^ = 13.080, *P* < .001). TNM staging was also related to the biological behavior of the tumor, with 9 of 32 (9/32, 28.1%) cases in stage I group, 23 of 35 (23/35, 65.7%) cases in stage II group, and 30 of 36 (30/36, 83.3%) cases in stage III group. The expression of MACC1 was positive in all 3 cases in stage Ⅳ, and there was a significant difference in the expression of MACC1 among the groups in TNM stage (*χ*^2^ = 24.399, *P* < .001). The above statistical analysis is shown in Table 3.

**Table 3:** Expression of MACC 1 in cervical squamous cell carcinoma and its correlation with clinicopathological factors.

Group	MACC1		*χ* ^2^	*P*
	Negative	Positive		
Age			0.124	.725
<60	15	26		
≥60	26	39		
Tumor size (cm)			0.951	.329
<4.0	21	27		
≥4.0	20	38		
Smoking			2.772	.100
No	34	47		
Yes	7	18		
Drink			0.001	1.000
No	27	43		
Yes	14	22		
Tumor grade			19.599	<.001
High	26	16		
Moderate	15	36		
Low	0	13		
Lymphatic metastasis			13.080	<.001
N0	35	33		
N1	6	32		
TNM stage			24.399	<.001
I	23	9		
II	12	23		
III	6	30		
IV	0	3		

MACC1 = colon cancer metastasis-associated protein 1, TNM = tumor-node metastasis.

#### 3.1.3. Correlation between the expression of AGR2 in tissues and clinicopathological factors

**Figure 3.**
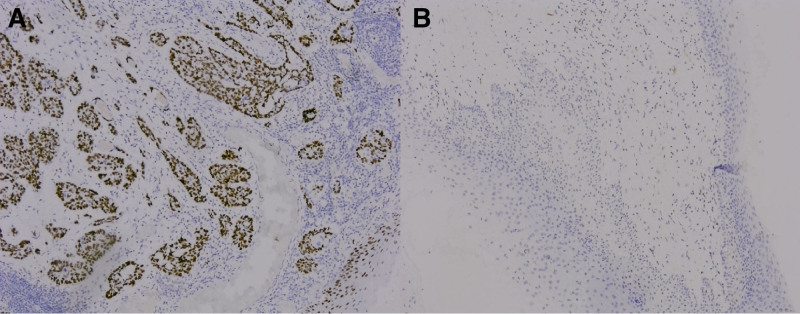
Expression of AGR2 in cervical squamous cell carcinoma and corresponding non-tumor control tissues. (A) Positive expression of AGR2 in cervical squamous cell carcinoma; (B) AGR2 is negatively expressed in normal squamous epithelium. AGR2 = anterior gradient-2.

Within a cohort of 106 patients diagnosed with squamous cell carcinoma of the cervix, 39 cases (39/106, 36.8%) exhibited positive AGR2 expression, while 20 cases (20/106, 18.9%) displayed positivity in the corresponding adjacent non-tumor tissues (*P* < .05). This variance in expression levels between the tumor cohort and the non-tumor control group was statistically significant (refer to Fig. 3A and B). In the subgroup of 41 patients under 60 years of age, 11 cases (11/41, 26.8%) tested positive for AGR2, whereas 28 out of 65 cases (28/65, 43.1%) in the older cohort (≥60 years of age) showed similar results. However, the difference in AGR2 expression between these 2 age groups was not statistically significant (*χ*^2^ = 2.854, *P* = .091). Additionally, among patients with tumor sizes <4.0 cm, 15 out of 48 (15/48, 31.3%) exhibited positive AGR2 expression, compared to 24 out of 58 (24/58, 41.4%) in those with tumors measuring 4.0 cm or greater. Again, no statistically significant difference was observed between these groups (*χ*^2^ = 1.159, *P* = .282). When considering smoking history, 29 out of 81 patients (29/81, 35.8%) in the nonsmoking group tested positive for AGR2, while 10 out of 25 patients (10/25, 40.0%) with a history of smoking also showed positive results; however, this difference was not statistically significant (*χ*^2^ = 0.304, *P* = .741). In terms of drinking history, positive AGR2 expression was found in 27 out of 70 patients (27/70, 38.6%) without a drinking background and in 12 out of 36 patients (12/36, 33.3%) with a drinking history, with no significant difference noted (*χ*^2^ = 0.154, *P* = .861). The analysis revealed a correlation between the biological behavior of the tumor and its differentiation grade. Specifically, AGR2 positivity varied across different differentiation levels, with 10 out of 42 (10/42, 23.8%) cases in the well-differentiated category, and 17 out of 51 (17/51, 33.3%) cases in the moderately differentiated category. Notably, the differential differentiation group exhibited a high AGR2 positivity rate of 12 out of 13 cases (12/13, 92.3%), indicating a significant difference in expression among these differentiation categories (*χ*^2^ = 20.535, *P* < .001). Lymph node metastasis (LNM) served as an indicator of the tumor’s malignant biological behavior, with positive AGR2 expression noted in 18 out of 68 patients (18/68, 26.5%) classified as N0 and in 21 out of 38 patients (21/38, 55.3%) classified as N1, demonstrating a significant difference in AGR2 expression relative to lymph node metastasis (*χ*^2^ = 9.138, *P* < .001). Finally, TNM staging emerged as a critical aspect of clinical evaluation, correlating with tumor biological behavior. Among 32 patients with stage I tumors, 3 cases were positive (3/32, 9.4%), in 35 cases of tumor stage II, 14 cases were positive (14/35, 40.0%), and in 36 cases of tumor stage III, 19 cases were positive (19/36), the expression of AGR2 was positive in 3 cases of stage IV, and there was a significant difference in the expression of AGR2 among the groups of TNM stage (*χ*^2^ = 19. 608, *P* < .001). The above statistical analysis is shown in Table 4.

**Table 4:** Expression of AGR2 in cervical squamous cell carcinoma and its correlation with clinicopathological factors.

Group	AGR2		*χ* ^2^	*P*
	Negative	Positive		
Age			2.854	.091
<60	30	11		
≥60	37	28		
Tumor size (cm)			1.159	.282
<4.0	33	15		
≥4.0	34	24		
Smoking			0.304	.741
No	52	29		
Yes	15	10		
Drink			0.154	.861
No	43	27		
Yes	24	12		
Tumor grade			20.535	<.001
High	32	10		
Moderate	34	17		
Low	1	12		
Lymphatic metastasis			9.138	<.001
N0	50	18		
N1	17	21		
TNM stage			19.608	<.001
I	29	3		
II	21	14		
III	17	19		
IV	0	3		

AGR2 = anterior gradient-2, TNM = tumor-node metastasis.

#### 3.1.4. Correlation of KAI1, MACC1, and AGR2 expression in cervical squamous cell carcinoma

Spearman correlation coefficient analysis showed that there was a negative correlation between KAI1 expression and MACC1 expression (*R* = −0.426, *P* < .001), and a negative correlation between KAI1 expression and AGR2 expression (*R* = −0.338, P *P* < .001); meanwhile, there was a positive correlation between MACC1 expression and AGR2 expression (*R* = 0.244, *P* = .012), as shown in Table 5.

**Table 5:** Correlations between KAI1, MACC1, and AGR2 expression in cervical squamous cell carcinoma.

Group	KAI1		*r*	*P*	MACC1		*r*	*P*
	Negative	Positive			Negative	Positive		
AGR2			-0.338	<.001			0.244	.012
Negative	32	35			32	35		
Positive	32	7			9	30		
MACC1			-0.426	<.001				
Negative	14	27						
Positive	50	15						

AGR2 = anterior gradient-2, KAI1 = anti-cancer 1, MACC1 = colon cancer metastasis-associated protein 1.

### 3.2. The expression of KAI1, MACC1, and AGR2 and the influence of clinicopathological factors on the survival time of patients

#### 3.2.1. Univariate and multivariate survival analysis

**Figure 4.**
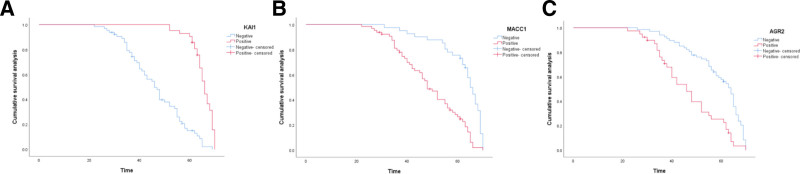
Survival curves of KAI1, MACC1, and AGR2. (A) Survival curves of KAI1; (B) survival curves of MACC1; (C) survival curves of AGR2. AGR2 = anterior gradient-2, KAI1 = anti-cancer 1, MACC1 = colon cancer metastasis-associated protein 1.

The impact of KAI1, MACC1, and AGR2 gene expression on postoperative survival rates among patients with cervical squamous cell carcinoma was assessed using the Kaplan–Meier method for univariate survival analysis. The findings indicated that patients exhibiting positive KAI1 expression had a mean survival duration of 65.0 ± 4.5 months, in contrast to those with negative KAI1 expression, whose survival time averaged 45.6 ± 11.6 months, Log-rank = 58.573, *P* < .001, and the difference was statistically significant, as shown in Table 6 and Figure 4A. The survival time of patients with positive MACC1 expression was 47.8 ± 12.5 months, which was significantly lower than that of patients with negative MACC1 expression was 61.9 ± 9.7 months, log-rank = 29.840, *P* < .001, and the difference was statistically significant, as shown in Table 6 and Figure 4B. The survival time of patients with AGR2 positive expression was 45.3 ± 13.0 months, which was significantly lower than that of patients with AGR2 negative expression (57.9 ± 11.3 months), log-rank = 20.746, *P* < .001, and the difference was statistically significant, as shown in Table 6 and Figure 4C.

**Table 6:** Effect of KAI1, MACC1, and AGR2 on postoperative survival time in patients with squamous cell carcinoma of the uterine cervix.

Group	Frequency (n)	Overall survival time (mo)	Log-rank	*P*
KAI1			58.573	<.001
Negative	64	45.6 ± 11.6		
Positive	42	65.0 ± 4.5		
MACC1			29.840	<.001
Negative	41	61.9 ± 9.7		
Positive	65	47.8 ± 12.5		
AGR2			20.746	<.001
Negative	67	57.9 ± 11.3		
Positive	39	45.3 ± 13.0		

AGR2 = anterior gradient-2, KAI1 = anti-cancer 1, MACC1 = colon cancer metastasis-associated protein 1.

The influence of clinicopathological factors on the postoperative survival time of patients with cervical squamous cell carcinoma: in terms of age, the postoperative survival time of patients <60 years old was 52.9 ± 13.6 months, and that of patients more than 60 years old was 53.5 ± 13.3 months, log-rank = 0.533, *P* = .465, the difference was not statistically significant; In terms of tumor size, the survival time of patients with tumor size < 4.0 cm was 55.7 ± 13.9 months, while that of patients with tumor size ≥ 4.0 cm was 51.2 ± 12.6 months, log-rank = 2.502, *P* = .114, the difference was not statistically significant. In terms of smoking history, the survival time of patients without smoking history was 55.0 ± 14.4 months, while the survival time of patients with smoking history was 52.2 ± 12.7 months, log-rank = 4.657, *P* = .051, the difference was not statistically significant. The survival time of patients with well-differentiated tumors was 59.1 ± 12.8 months, the survival time of patients with moderately differentiated tumors was 52.1 ± 11.5 months, and the survival time of patients with poorly differentiated tumors was 39.2 ± 11.0 months, log-rank = 26.879, *P* < .001, the difference was statistically significant. In terms of lymph node metastasis factors, the survival time of N0 grade tumor patients was 60.5 ± 8.8 months, while that of N1 grade tumor patients was 41.5 ± 9.2 months, log-rank = 161.345, *P* < .001, the difference was statistically significant. In TNM staging, the survival time of patients with tumor stage I was 66.1 ± 3.1 months, the survival time of patients with tumor stage II was 56.3 ± 8.1 months, the survival time of patients with tumor stage III was 41.4 ± 9.4 months, and the survival time of patients with tumor stage IV was 26.3 ± 4.0 months, log-rank = 176.566. The difference was statistically significant (*P* < .001). See Table 7 for details.

**Table 7:** Influence of clinicopathological factors in cervical squamous cell carcinoma.

Group	Frequency (n)	Overall survival time (mo)	Log-rank	*P*
Age			0.533	.465
<60	41	52.9 ± 13.6		
≥60	65	53.5 ± 13.3		
Tumor size (cm)			2.502	.114
<4.0	48	55.7 ± 13.9		
≥4.0	58	51.2 ± 12.6		
Smoking			4.657	.051
No	81	55.0 ± 14.4		
Yes	25	52.2 ± 12.7		
Drink			0.750	.387
No	70	54.9 ± 12.8		
Yes	36	52.0 ± 13.8		
Tumor grade			26.879	<.001
High	42	59.1 ± 12.8		
Moderate	51	52.1 ± 11.5		
Low	13	39.2 ± 11.0		
Lymphatic metastasis			161.345	<.001
N0	68	60.5 ± 8.8		
N1	38	41.5 ± 9.2		
TNM stage			176.566	<.001
I	32	66.1 ± 3.1		
II	35	56.3 ± 8.1		
III	36	41.4 ± 9.4		
IV	3	26.3 ± 4.0		

TNM = tumor-node metastasis.

Multivariate COX regression analysis showed that the positive expression of KAI1, MACC1, and AGR2, TNM stage and lymph node metastasis stage in clinicopathological factors were independent prognostic factors for the OS time of patients with cervical squamous cell carcinoma (*P* < .05). See Table 8 for details.

**Table 8:** Results of the multivariate postoperative survival analysis.

Group	B	SE	*P*	HR	95% CI
LNM	1.161	0.520	.026	3.193	1.152–8.846
TNM stage	0.568	0.289	.049	1.765	1.002–3.110
KAI1	-1.339	0.319	<.001	0.262	0.140–0.490
MACC1	0.797	0.281	.005	2.219	1.280–3.848
AGR2	0.736	0.287	.010	2.088	1.896–3.668

AGR2 = anterior gradient-2, KAI1 = anti-cancer 1, LNM = lymph node metastasis, MACC1 = colon cancer metastasis-associated protein 1, TNM = tumor-node metastasis.

## 4. Discussion

Cervical squamous cell carcinoma exhibits significant heterogeneity, which may compromise the reliability of biomarker evaluations. Consequently, a comprehensive assessment of the role of biomarkers in tumor prognosis and metastasis is essential to validate their efficacy. The primary factor contributing to the inadequacy of cancer treatment is the metastasis and recurrence of tumors. Therefore, it is imperative to identify novel and more effective biomarkers for the assessment and prediction of patient prognosis.

The inactivation of genes that suppress tumor metastasis is crucial in the progression of metastatic tumor cells. KAI1, recognized as a tumor metastasis suppressor gene, is known to impede tumor cell metastasis by either enhancing intercellular adhesion or diminishing the adhesion between cells and the extracellular matrix.^[19]^ In our investigation, we observed that the positive expression rate of KAI1 in cervical squamous cell carcinoma tissues was markedly lower compared to that in the corresponding non-tumor control tissues. Furthermore, this positive expression rate exhibited a negative correlation with various parameters, including tumor size, histological grade, TNM stage, and LNM stage. Moreover, Kaplan–Meier univariate survival analysis indicated that patients with KAI1-positive cervical squamous cell carcinoma experienced a significantly longer OS compared to those with KAI1-negative tumors. These results imply that the down-regulation or complete loss of KAI1 expression may facilitate the advancement and metastasis of tumor cells, consequently leading to a reduction in postoperative survival time and adversely affecting patient prognosis. These observations align closely with findings from earlier studies.^[20,21]^

MACC1 has emerged as a potential biomarker that could serve as a valuable predictor for tumor metastasis and patient prognosis across a spectrum of cancers. Research indicates that MACC1 plays a crucial role in several fundamental biological processes associated with tumors, including tumorigenesis, proliferation, invasion, metastasis, and EMT.^[22,23]^ In our investigation, we observed that the expression levels of MACC1 in cervical squamous cell carcinoma tissues were markedly elevated compared to those in the corresponding control tissues. Furthermore, we discovered that the overexpression of MACC1 exhibited a positive correlation with tumor histological grade, TNM staging, and LNM staging. Our univariate survival analysis revealed that the OS duration of patients with MACC1-positive cervical squamous cell carcinoma was significantly shorter than that of their MACC1-negative counterparts. These findings align with those reported in prior studies,^[24,25]^ reinforcing the notion that MACC1 may serve as a valuable biological marker for cervical squamous cell carcinoma.

EMT is integral to the development of normal tissues, the inflammatory response, and the metastatic behavior of tumor cells. Numerous investigations have indicated that AGR2 overexpression is associated with the EMT process within neoplastic tissues.^[26,27]^ Furthermore, heightened levels of AGR2 may facilitate tumor cell proliferation and metastasis, consequently exerting detrimental effects on clinical treatment outcomes.^[28,29]^ In our research, we observed that the expression levels of AGR2 in cervical squamous cell carcinoma tissues were markedly elevated compared to their corresponding non-neoplastic control tissues. Moreover, we identified a positive correlation between AGR2 overexpression and tumor grade, TNM stage and LNM stage. In line with findings regarding MACC1, patients diagnosed with AGR2-positive squamous cell carcinoma of the cervix exhibited significantly shorter OS times than those with AGR2-negative tumors. Comparable findings have been documented in other research, underscoring the close association between AGR2 overexpression and tumor progression as well as metastasis.^[30,31]^

In this investigation, a multivariate COX regression survival analysis revealed that the positive expression of KAI1, MACC1, and AGR2, along with TNM stage and LNM stage, are independent prognostic indicators for survival in individuals diagnosed with cervical squamous cell carcinoma. Consequently, our findings imply that KAI1, MACC1, and AGR2 should be regarded as valuable biomarkers for this malignancy, particularly in the context of predicting tumor metastasis and patient outcomes.

It is widely acknowledged that the metastatic spread of tumor cells encompasses a series of intricate mechanisms, including the deactivation of tumor metastasis suppressor genes, the activation of tumor metastasis-promoting factors, and the process of EMT. MACC1 has been shown to enhance tumor cell proliferation and metastasis via the activation of the HGF/C-MET signaling pathway.^[32,33]^ In our study, we observed a negative correlation between the expressions of MACC1 and KAI1. Prior research has indicated that KAI1 can exert its biological effects by forming a complex with C-MET or by inhibiting HGF activation.^[34]^ Specifically, KAI1 can suppress MACC1 activation, thereby hindering the migration and metastasis of tumor cells.^[35]^ Furthermore, KAI1 expression was also found to be inversely related to AGR2 expression. The overexpression of AGR2 is known to facilitate tumor cell proliferation, invasion, metastasis, and EMT. Adequate levels of KAI1 can counteract EMT in tumor cells by enhancing the formation of theβ-catenin/E-cadherin complex.^[36]^

Conversely, abnormal KAI1 expression may result in the loss of EMT inhibition, consequently fostering tumor cell invasion and metastasis. Additionally, the expressions of MACC1 and AGR2 were positively correlated, with their overexpression collaboratively driving tumor cell proliferation, invasion, metastasis, and EMT. The interplay between KAI1, MACC1, and AGR2 was associated with postoperative survival duration, and their combined assessment could serve as a prognostic predictor for patients.

## Acknowledgments

We thank all colleagues in the ChangZhou Maternal and Child Health Hospital Affiliated to Nanjing Medical University, for their help and support in this study.

## Author contributions

**Conceptualization:** Pengfeng Zhu.

**Data curation:** Pengfeng Zhu.

**Formal analysis:** Pengfeng Zhu.

**Funding acquisition:** Pengfeng Zhu.

**Investigation:** Pengfeng Zhu.

**Resources:** Siyuan Wang.

**Software:** Siyuan Wang.

**Supervision:** Siyuan Wang.

**Validation:** Siyuan Wang.

**Writing – original draft:** Siyuan Wang.

**Writing – review & editing:** Siyuan Wang.
